# Office Notes—Destroying Nerves

**Published:** 1872-03

**Authors:** W. H. Shadoan


### OFFICE NOTES—DESTROYING NERVES.

BY W. H. SHADOAN, D. D. S.

This operation is condemned by a few operators, as wholly unnecessary. We will agree that many nerves are destroyed that should have been preserved, but there are cases in which I do not attempt to save pulps.

The question under consideration is not when a pulp should, or should not be destroyed, but the easiest way of destroying it and causing the least pain to the patient.

*Preparation of case.*—The nerve should in all cases be laid bare before the application is made, otherwise there is a liability of great pain for a time at least. It is not always that the patient can bear the pain of the operation; when this is so, the application of chloroform for a short time, or creosote and tincture aconite will obtund the sensibility until the excavation can be completed.

If the tooth to be operated on has but a single root, the point of exposure need not be so large; half a line in diameter will do; but if it is a molar, then always make the orifice of exposure as large as possible. A full line in diameter, if circumstances will permit. Wound the pulp, if possible, before its excavation. A drop of blood from the nerve of a tooth depletes it sufficiently to allow of the destruction with rarely ever any pain, and after all, that is just what is desirable.

*Material Used.*—I invariably use *arsenious acid* and *carbolic acid* without sulphate or the acetate of morphia. I once used a paste of arsenious acid and sulph morphia, and in nine cases out of ten there was great pain for a considerable time, hence its total abandonment. Now I use only arsenic and carbolic acid.

*Method of Application.*—It matters very little where the application is to be made: whether in an incisor tooth or a third molar, the tooth should be invested with a napkin; dry out the cavity thoroughly with punk, or softly rolled bibulous paper, and with carbolic acid wet the pulp and surrounding portion of the dentine; then the arsenic is applied on the point of a small bit of cotton rolled between the fingers until it is quite firm about a line in diameter, and as long as necessary. (For a front tooth a quarter of an inch, and for a back tooth a half of an inch long, will be required.) This is taken up with the plugging pliers, and dipped into the solution of acid, and the point touched to the dry pulverized arsenic, when a sufficient amount will adhere to the cotton, which is carried directly to the point intended. In this way the application may be made directly on the point of exposure, and never allowed to come in contact with the gum or lip of the patient. A piece of cotton laid on the arsenic loosely is all that is needed to complete the operation. I never leave the cotton used to carry the arsenic to the nerve in the tooth, unless it be a molar, and seldom then. The arsenic should be removed within twenty-four hours, when generally the nerve is sufficiently obtunded to be removed, though frequently the pulp may remain for a few days without molestation, when it can more easily be removed.

### AN UGLY CASE.

A lad twelve years of age was brought by his father for an examination of his teeth. The examination disclosed several small cavities and one very large.

An appointment was made for a sitting, at which time the operation was commenced, and the case proved the most intractible one I ever had. The first difficulty was a small mouth, which, when fully opened, did not expose the teeth beyond the first bicuspid. The second difficulty was the extreme sensitiveness of the dentine. The location of the decay would of itself be sufficient to excite suspicion that something was wrong.

The first right sup. molar had three cavities in it, one in the anterior and one in the posterior crown pits, and the other in the posterior lingual cusp on the slope facing the anterior pit and greatest prominence of the cusp. The first right inferior molar, anterior buccal cusp was decayed in center. The central slope of the anterior lingual cusp was also decayed, as well as two other points in the masticating surface of the same tooth. This tooth was decayed on the most prominent points, while the pits which were somewhat deep were free from decay. The corresponding tooth on the opposite side of the mouth is decaying in the same manner as the one just named, but not so badly.

The second left superior bicuspid was largely decayed in the posterior approximal surface, also antero-buccal surface midway between gum and grinding surface; first left sup molar giving away very much as also the first right molar. I have thus given the location of the decays, none of which was larger within than on the surface, except the first cavity named, and the posterior proximal of second bicuspid, which latter extended quite up to the nerve. The sensitive condition of the dentine rendered it almost impossible to excavate the cavities without causing the most intense pain. Another difficulty was the impossibility of keeping the tongue still while operating, and lastly, though not the least, was the tendency to suffocation whenever anything was put in the mouth to protect the cavities while filling. To attempt to adjust and keep the coffer dam in place was utterly impossible. I have filled, or "filled at," those needing the most immediate attention, but I am free to say if all my patients were like this one, I should feel inclined to change my occupation, much as I love the profession.

TO BE CONTINUED.

